# The “H sign” in MOGAD myelitis

**DOI:** 10.1007/s10072-025-08160-4

**Published:** 2025-05-06

**Authors:** Santiago Aristizabal Ortiz, Laura Andrea Campaña Perilla, Angela Patricia Guarnizo Capera

**Affiliations:** 1 Department of Diagnostic Imaging, Fundación Santa Fe de Bogotá, 116 street # 9-02, Bogotá, 110111 Colombia; 2 School of Medicine, Specialization in Radiology and Diagnostic Imaging, Universidad del Bosque., Bogotá, Colombia

**Keywords:** Demyelinating diseases, MOGAD, Myelin oligodendrocyte glycoprotein antibody–associated disease, Magnetic Resonance Imaging

## Abstract

A 40-year-old man presented with acute urinary retention and bilateral lower limb weakness. MRI revealed a longitudinally extensive T2 hyperintense spinal cord lesion from C6 to T10, predominantly involving the gray matter (“H sign”). Brain MRI and CSF studies were unremarkable. Serum MOG-IgG was positive. The patient was treated with intravenous corticosteroids, with subsequent clinical and radiologic improvement. The imaging and clinical findings were consistent with myelin oligodendrocyte glycoprotein antibody-associated disease (MOGAD).

## Case presentation

**Fig. 1:**
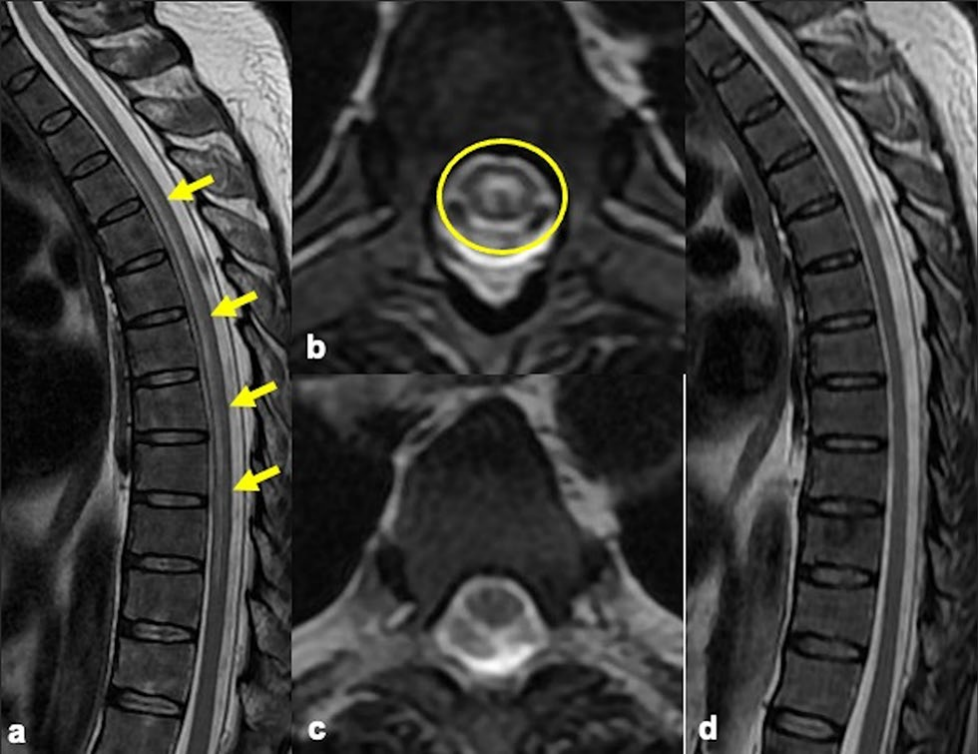
Sagittal and axial T2 MRI weighted images (**a**, **b**) show longitudinally extensive myelitis (arrows) involving the central gray matter with an “H shape” appearance on axial images (circle). Axial and sagittal T2 follow-up MRI (**c**, **d**) demonstrates resolution of the abnormal high T2 signal

A 40-year-old man with a prior history of leukemia, presented to the emergency department with 1 day of impaired mobility and urinary retention. Physical examination revealed diffuse severe weakness in his lower extremities and abnormal reflexes (3+). Cervical and thoracic spine MRI demonstrated abnormal high T2 cord signal extending from C6 to T10 without cord edema or enhancement (Fig. 1). Brain MRI (not shown) was unremarkable. CSF analysis was negative for virus, bacteria, fungus, and tumor markers. Oligoclonal bands and aquaporin-4 antibodies were absent. Serum MOG- IgG testing was positive. The patient was treated with high-dose intravenous corticosteroids and discharged to a rehabilitation unit. On follow-up, the patient’s symptoms improved, and the spine MRI showed resolution of the longitudinally extensive myelopathy (Fig. 1).

## Discussion

Myelin oligodendrocyte glycoprotein antibody-associated disease (MOGAD) is a distinct neuroinflammatory disease with MOG-IgG seropositivity that presents unique clinical, paraclinical, and imagenological features [1]. Its pathophysiology remains unclear but likely relates to MOG-IgG antibodies to the MOG protein, a myelin protein in the outer myelin sheath layers, and the cell surface of oligodendrocytes [2]. The disease has a female-to-male ratio of 1:1 and can present at any age but its frequency seems higher in children [2]. Typically infectious prodrome precedes the onset of the symptoms [1].

After anterior segment optic neuritis, longitudinally extensive myelitis is another common presentation of MOGAD. T2 hyperintensities affect both gray and white matter with greater than 50% involvement in the axial Sects. [1, 2]. In cases of isolated gray matter involvement, the appearance on axial images resembles an H (“H” sign), a finding seen in 30 − 50% of MOGAD patients, with much lesser extent in AQP4-IgG positive NMOSD and absent in multiple sclerosis (MS) patients. This finding is suggestive of gray matter inflammation.

T2 hyperintense short lesions smaller than 2 vertebral bodies have also been described and in these cases, MS must be considered in the differential. Furthermore, high T2 signal lesions usually affect the conus in a greater extent compared to AQP4-IgG positive NMOSD, and it is considered highly specific for MOGAD diagnosis [3]. Cord lesions enhancement can be seen in 50% of the cases and it is usually less avid than in NMOSD and MS [3].

In conclusion the “H sign” represents spinal cord myelitis isolated to the gray matter. This sign, although not specific, should raise the suspicion of MOGAD and together with other typical imaging features and clinical/paraclinical findings can further reduce the differential.
